# Global bioethics and moral pluralism: towards a criteriology at the hospital

**DOI:** 10.1186/s12967-024-05440-z

**Published:** 2024-07-04

**Authors:** Henri-Corto Stoeklé, Christian Hervé

**Affiliations:** 1 Department of Ethics and Scientific Integrity, Foch Hospital, 40 rue Worth, Suresnes, 92150 France; 2 Medical School, Paris Cité University, Paris, France; 3 Department of One Health and Global Health, Medical School, Versailles Saint-Quentin-en- Yvelines University (UVSQ), Montigny-le-Bretonneux, France

**To the editor**

As we published in the *Journal of Translational Medicine* in 2023 [1], we have begun to apply in our recent studies in empirical bioethics a particular ethical theory: ‘global bioethics’. However, we have also begun to rework this teleological theory to provide, more formally, greater operationality for the resolution of ‘bio-ethical issues’(i.e. tensions between medical and/or biological practices and moral values and/or standards), pre-identified by various methodological approaches (e.g. qualitative approach, etc.), at a global scale (i.e. ‘macro-bio-ethical issues’; e.g. country, etc.), and/or local scale (i.e. ‘micro-bio-ethical issues’; e.g. institution, etc.), and then resolved by this theory, as a function of the real and/or potential consequences for ‘survival’ and/or ‘happiness’, at individual and/or collective levels. Practical limitations have nevertheless emerged in the face of ‘moral pluralism’ (i.e. the presence of several moral frameworks) , in our case, at the hospital.

Worldwide, this pluralism results from the existence of ‘cultural areas’ that differ to various extents. These spaces are delimited to various degrees, and at a given point in time present cultures displaying various degrees of difference, or homogeneity. At the regional scale, phenomena relating to multiculturalism may also apply, themselves emanating from other complex phenomena (e.g. human migration, etc.). In hospitals, and elsewhere, as we have ourselves observed, this pluralism influences the likelihood of medical and/or biological practices being accepted, or refused, by individuals, or groups of people [2]. This pluralism must be better taken into account in global bioethics, as a leading ethical theory, to increase the operationality of this theory and, thus, its practical pertinence, especially at the hospital.

In this theory, our first postulate is that survival and happiness are the only two legitimate ‘bio-ethical objectives’ common to the whole of humanity, consciously or otherwise. Our second postulate is that these objectives are wholly or partially, and explicitly or implicitly, conditioned by different cardinal criteria correlated with these different moral frames of reference of individuals and/or society [3]. We then began to characterize these frameworks, as a means of outlining these criteria, by analyzing the content of a collective work by colleagues, which remains a reference in this domain within the French-speaking world [4]. Here, we present a summary of this analysis and its perspectives, a draft of which we have formulated in French in the chapter of another collective work [5].

We first manually identified and listed in alphabetical order 12 principal moral frameworks, which we developed into the corresponding number of chapters: the ‘Animist framework’, the ‘Buddhist framework’, the ‘Catholic framework’, the ‘Confucianist framework’, the ‘Hindu framework’, the ‘Islamic framework’, the ‘Jewish framework’, the ‘Kantian framework’, the ‘lay framework’, the ‘Orthodox framework’, the ‘Protestant framework’ and the ‘Utilitarian framework’. We then characterized, manually again, the basis, aims and rules of each framework, through excerpts that appeared significant, which we sometimes rearranged, from which we induced these criteria. These frameworks and criteria are non-exhaustive, and they are collated in a detailed table for greater clarity (N.B. see the table).

**Table 1:** Graphical summary of the analysis of content

Moral pluralism					
Frameworks	Chapters	Basis	Aims	Rules	Criteria
Animist	L. M. Paomé, “Éthique animiste,” Chap. 7 in *Nouvelle encyclopédie de bioéthique: médecine, environnement, biotechnologie* (De Boeck Université 2001): 56–61.	*“The life force* […] *and a ranking of life forces* […] *At the summit, we find the breath of divine life, then the life force of ancestors, geniuses* […] *After these invisible beings, the life force passes through the heads of clans and villages, and then other humans, finally dispersing in animals, plants and things”*	“*Group survival*”	“*Respect of ancestors and of the prohibitions they dictated, respect for the elderly and for all nature* […] *Solidarity of the living with the dead and between living beings*”	Life forces, ancestors, group etc.
Buddhist	J. Martin, “Bioéthique et bouddhisme,” Chap. 35 in *Nouvelle encyclopédie de bioéthique: médecine, environnement, biotechnologie* (De Boeck Université 2001): 149 − 51.	“*The supreme holiness of human life and of all forms of existence in general* […] [But] *the supreme holiness of human life is the most important* […] [Because] *human life is very difficult to acquire in the Buddhist approach and only human life can deliver us from the cycle of existence*”	“*The perfect and definitive beatitude that accompanies this deliverance*”	“*Abstention from negative acts* […] [that] *keep their author in the painful cycle of existences, whereas positive acts* […] *create the conditions for a happy life and lead to deliverance from the cycle of existence* […] *Through practices of concentration and penetrating vision*”	Soul, reincarnation, life in general, etc.
Catholic	B. Cadoré, “Bioéthique et catholicisme,” Chap. 39 in *Nouvelle encyclopédie de bioéthique: médecine, environnement, biotechnologie* (De Boeck Université 2001): 162-9.	“*A human person* [who] *is considered as his/her interior unit, that by which the physical, psychic and spiritual dimensions are integrated* […] [And the] *notion of dignity*, [which] *is linked to the recognition of a relationship between man, created in the image of God, and his creator*”	“*Respect for the dignity and right to life of all human beings*”	“*The value of the person, everyone needing to be recognized for themselves and in an unconditional manner, cannot be subordinated to aims other than their personal being* […] *Moral standards protect this value of the human person”*	God, the human person, dignity of the person, etc.
Confucianist	B. Leclerc, “Bioéthique et confucianisme,” Chap. 56 in *Nouvelle encyclopédie de bioéthique: médecine, environnement, biotechnologie* (De Boeck Université 2001): 223-6.	“*Relationships.* [Because] *man cannot conceive of himself outside of his natural and social environment* […] [And] *the indissociable order* […] *the notion of change* […] [which] *is understood* [i.e. order] *from the concomitance of phenomena that succeed each other regularly over the course of time* […] *The notion of change* [that] *sees in this cyclic series a relationship of creation that guarantees the long-term existence of the world*”	“*Man of quality* […] *The search for harmony that he finds in social relations*”	“*Introspection, the culture of emptiness* […] *Study, which, above all, takes the form of learning to live*”	Harmony, social and natural environment, man of quality, etc.
Hindu	J. Azariah, “Bioéthique et hindouisme,” Chap. 135 in *Nouvelle encyclopédie de bioéthique: médecine, environnement, biotechnologie* (De Boeck Université 2001): 496–504.	“*A well-coordinated system of beliefs* […] [Firstly] *human beings and nature were never envisaged as separate from each other* […] *The question of exploiting nature is unimaginable* […] [Secondly] *the division of society into castes* […] [to which] *it is a duty to be attached*”	“*Liberate the soul* [i.e. that of a Hindu] *from earthly suffering* […] [Because] *the greatest good in life is to liberate it from the constraints of the flesh, to be in a state of complete and absolute cessation of all suffering and corporality*”	“*The way of work* […] *the way of prayer* […] *the way of knowledge*”	Soul, nature, castes, etc.
Islamic	F. Haddad-Chamakh et al., “Bioéthique et islam,” Chap. 148 in *Nouvelle encyclopédie de bioéthique: médecine, environnement, biotechnologie* (De Boeck Université 2001): 545-8.	“*The order of institutional structures of kinship, the framework of the social cement as decreed by God in the eyes of believers*”	“*The idea of a worthy life* […] *of an unequivocal and theologically founded way, the lines of demarcation between the licit and illicit*”	“*Fiqh (jurisprudence)* […] *a sort of methodology with well-fixed standards, enabling jurists to instruct judgments on a multitude of cases of new types that arise, using rules of analogy and deduction, thereby guaranteeing that all possible instances of jurisprudence are logically connected to the principles of the dogma* […] *and to the initial precepts of the divine word revealed*”	God, family, *Fiqh*, etc.
Jewish	A. Guigui, “Bioéthique et judaïsme,” Chap. 149 in *Nouvelle encyclopédie de bioéthique: médecine, environnement, biotechnologie* (De Boeck Université 2001): 549 − 54.	“*On the written (the Pentateuch) and oral (the Talmud) texts of the Torah. It refers to the commandments regulating the relationship of humans with each other and the relationship of humans with God.* [But] *Jewish law is not static. It evolves*”	“*Respect for human life* [which] *is absolute, sacred, inviolable* […] *Because man was made in the image of God* […] *However, it is more important to save the group than to save the individual*”	“*Religious prescriptions* [that] *are modified or even entirely ignored to save a life in danger* […] [Because] *saving a human life is more important than the study of the Torah*”	Torah, human life, collectivity, etc.
Kantian	E. Schroten, “Éthique kantienne,” Chap. 151 in *Nouvelle encyclopédie de bioéthique: médecine, environnement, biotechnologie* (De Boeck Université 2001): 677 − 80.	“*Equal humanity* […] [or] *dignity in everyone*”	“*Do not deal with humanity in oneself and in others only as a means, but also as an end*”	“*Autonomy, like the responsibility of each of us to find, by ourselves, for each concrete situation, by exploring that situation in depth, the derived maxims, intermediary principles, conditioned rights and, thus, the right measure for their reciprocal resistance that will best concretize, that is, in the manner best adjusted to the conditions, the unconditioned form of the imperative* […] , *which is nothing other than the principle of respect for the equal humanity in each of us*”	Humanity, autonomy, Kantian imperative, etc.
Lay	H. T. Engelhardt and A. S. Iltis “Bioéthique et laïcité,” Chap. 153 in *Nouvelle encyclopédie de bioéthique: médecine, environnement, biotechnologie* (De Boeck Université 2001): 567-8.	“*A liberal cosmopolitan ethos including a post-Christian affirmation of individual liberty* […] [And] *the collapse and/or marginalization of cultural sources, traditionally religious and professional guidelines*”	“*Giving meaning to reproduction, birth, life, health and disease, suffering and death in universal terms, that is, terms that are not historical, cultural or religious*”	“*Autonomous choice of individual moral agents* [who] *by default, occupy the center stage.* [Because] *if we cannot call on divine guidance or the standardization of professional ethics or a particular cultural perspective, then the individual choices of human actors become capital*”	Liberty, moral agent, autonomous choice, etc.
Orthodox	J. Larchet, “Bioéthique et christianisme orthodoxe,” Chap. 176 in *Nouvelle encyclopédie de bioéthique: médecine, environnement, biotechnologie* (De Boeck Université 2001): 627 − 63.	“*The absolute value of each human being* [who] *is part of God*”	“*Every person must, thus, in all the dimensions of their being, nature and own identity, regardless of age or state, be the subject of absolute respect*”	“*Rules defined by religious councils, in the form of canons*, [but] *the entire collection of these canons does not, strictly speaking, form a ‘canonical law’ and their strict definition in terms of principles (or akribeia) is never without a certain flexibility of application to particular situations and personal cases (or economy)*”	God, canons, human beings, etc.
Protestant	E. Schroten, “Bioéthique et protestantisme,” Chap. 194 in *Nouvelle encyclopédie de bioéthique: médecine, environnement, biotechnologie* (De Boeck Université 2001): 677 − 80.	*“The belief that humans (sinners) are justified by the grace of God, placing them in a state in which they can accomplish the will of God, and thus, good. The justification of one’s existence (as a moral subject) is not controlled by humans through their own actions (assisted by the grace of God) but is obtained exclusively by the grace of God (sola gratia)”*	“*Moral responsibility of the believer […] to alert and assist believers to an enlightened personal moral responsibility*”	*“Evangelical passage […] The life of Christ […] A transition from attention to natural law towards the teaching and existence of Jesus Christ and, thus, the Bible”*	Bible, life of Christ, individual responsibility, etc.
Utilitarian	J. Goffi, “Éthique utilitariste,” Chap. 243 in *Nouvelle encyclopédie de bioéthique: médecine, environnement, biotechnologie* (De Boeck Université 2001): 853-7.	“*Consequentialist ethics*, [for which] *certain states of the world are considered good or desirable in themselves: these states constitute Good.* […] *The specificity of utilitarianism lies in its definition of Good as happiness and its consideration of happiness as a measurable entity. The different states of the world can be compared in terms of the amount of happiness found there*”	“*The greatest happiness of the largest number of people, which constitutes the measurement of justice and injustice*”	“*Principle of utility: the best action is that which procures the greatest happiness for the largest number of people* […] [and] *an extension of the moral community*”	Happiness, principle of utility, moral community, etc.

We can distinguish several tendencies from this table. The Catholic, Islamic, Jewish, Kantian, lay, Orthodox, Protestant and Utilitarian frameworks can be grouped together, contrasting with a second group of frameworks consisting of the Buddhist, Confucianist and Hindu frameworks. In the first group of frameworks, humans appear to be dissociated from nature, for various spiritual, religious and/or philosophical reasons, whereas this does not appear to be the case for the second group of frameworks. In addition, for similar reasons, the individual is generally more important than the collective in the first group of frameworks, although this is debatable for the Islamic, Jewish and Utilitarian frameworks, whereas the opposite is true for the second group of frameworks. The Animist framework, which is certainly more unique, converges more with the Buddhist, Confucianist and Hindu frameworks.

These frameworks and the criteria obtained through their analysis clearly have their limitations. Firstly, they are based on the contents of a single book. Secondly, a synthetic exercise of this type necessarily leads to simplifications, particularly for the Animist and Protestant frameworks, which are very heterogeneous. These frameworks and criteria are, thus, neither exhaustive nor definitive. As such, they need to be expanded by more empirical studies. They nevertheless provide indications, and constitute a preliminary basis for a criteriology that will eventually make it possible to render global bioethics more operational, particularly in hospitals. Furthermore, this ‘new’ global bioethics could better incorporate emerging paradigms such as ‘global health’ and ‘one health’.

Implicitly, these paradigms are included in global bioethics right from the start, because they fully condition our survival — and our happiness. However, they were included in an initial approach that we consider to be insufficiently pluralist and pragmatist in that, retrospectively, this approach appears to be too secular and materialist. In other words, there is an excessive expression of political and philosophical systems, essentially linked to the lay framework that is proving difficult to apply beyond the confines of the West. The survival and happiness of one set of people are not always those of another, which is why a criteriology such as that proposed here is useful. However, this criteriology should not favor a certain moral relativism, particularly as concerns racism, sexism or homophobia.

## Data Availability

Not applicable.

## References

[1] Stoeklé HC, Ivasilevitch A, Hervé C (2023). Good practice in medicine and biology: scientific integrity needs global bioethics. J Transl Med.

[2] Stoeklé HC, Sekkate S, Ayoubi JM, Bennouna J, Beuzeboc P, Hervé C (2023). Vaccination against HPV: Easier said than done?. Hum Vaccin Immunother.

[3] Stoekle HC, Marignac G, Hervé C. Macro-bio-ethical versus micro-bio-ethical issues concerning human brain organoids. AJOB Neurosci. 2023. 10.1080/21507740.2023.2188298 37097845

[4] Hottois G, Missa JN. Nouvelle encyclopédie de bioéthique: médecine, environnement, biotechnologie. De Boeck Université; 2001.

[5] Stoekle HC, Hervé C. Bioéthique globale appliquée: entre une (seule) santé Globale et un pluralisme moral. La Vie, le corps et la mort Réflexions Juridique et éthique. Dalloz; 2023. pp. 189–200.

